# Associating Flexible Regulation of Emotional Expression With Psychopathological Symptoms

**DOI:** 10.3389/fnbeh.2022.924305

**Published:** 2022-06-27

**Authors:** Gabriel Gonzalez-Escamilla, Denise Dörfel, Miriam Becke, Janina Trefz, George A. Bonanno, Sergiu Groppa

**Affiliations:** ^1^Department of Neurology, University Medical Center of the Johannes Gutenberg University Mainz, Mainz, Germany; ^2^Differential and Personality Psychology, Faculty of Psychology, Technische Universität Dresden, Dresden, Germany; ^3^Department of Clinical Psychology, Teachers College, Columbia University, New York, NY, United States

**Keywords:** emotion regulation, expressive flexibility, suppression (psychology), affective symptoms, psychopathology (mostly depressive disorders), mental health—related quality of life

## Abstract

**Background**: Stressful situations and psychopathology symptoms (e.g., depression and anxiety) shape how individuals regulate and respond to others’ emotions. However, how emotional expressions influence mental health and impact intrapersonal and interpersonal experiences is still unclear.

**Objective**: Here, we used the Flexible Regulation of Emotional Expression (FREE) scale to explore the relationship between emotional expression abilities with affective symptoms and mental health markers.

**Methods**: From a sample of 351 participants, we firstly validate a German version of the FREE scale on a final sample of 222 participants located in Germany, recruited through an online platform. Following this, we performed confirmatory factor analyses to assess the model structure of the FREE-scale. We then utilize a LASSO regression to determine which indicators of psychopathology symptoms and mental health are related to emotional expressive regulation and determine their particular interactions through the general linear model.

**Results**: We replicated the FREE scale’s four latent factors (i.e., ability to enhance and suppress positive as well as negative emotional expressions). After the selection of relevant instruments through LASSO regression, the suppress ability showed specific negative associations with depression (*r* = 0.2) and stress symptoms (*r* = 0.16) and positive associations with readiness to confront distressing situations (*r* = 0.25), self-support (*r* = 0.2), and tolerance of emotions (*r* = 0.2). Both, emotional expressions enhance and suppress abilities positively associated with coping markers (resilience) and emotion regulation skills. Finally, the interaction effects between emotional flexibility abilities and stress, depression, and anxiety symptoms evidenced that consistent with the flexibility theory, enhancing and suppressing abilities may predict psychopathological symptoms.

**Conclusions**: These findings emphasize the importance of considering the flexibility to express emotions as a relevant factor for preserved mental health or the development of psychopathological symptoms and indicate that online surveys may serve as a reliable indicator of mental health.

## Introduction

Human emotions fulfill important adaptive functions, for example in decision making, memory processes, or social interactions (Nelis et al., 2011; Gross, 2015). Emotions help us become aware of our goals, help us reach these goals, and provide information about threats to goal attainment. At the same time, however, emotions can lead to failure in goal achievement, for instance when the activated emotion prevents adaptation to the situation (Frijda, 2007). If the frequency, intensity, or type of emotion does not fit a given situation, emotions may become dysfunctional and interfere with personal goal attainments. This, thus, leads to the need to effectively regulate own emotions (Aldao, 2013; Aldao et al., 2015; Gross, 2015). In this regard, Gross’ (Extended) Process Model of Emotion Regulation (Gross, 1998, 2015) defines emotion regulation as the activation of a goal to modify an unfolding emotional response and the initiation of processes that change emotional experiences, expressions, and/or physiological responses. An emotion-generating process is perceived and action impulses are activated to modify this process using regulatory strategies according to its negative or positive evaluation. Thus, successful emotion regulation is characterized by appropriate emotional awareness, emotion-regulation goals, and emotion-regulation strategies (Gross and Jazaieri, 2014).

Emotion-regulation goals can be manifold (Tamir, 2009). Although, traditionally, research focused on the downregulation of negative emotions (referring to schemes to decrease and diminish the intensity of an emotional experience, and minimize behavioral and even facial responses) and/or the up-regulation of positive emotions (representing the hedonic goal “I want to feel better.”), the down-regulation of positive or the upregulation of negative emotions (as a means to another goal, for instance, “I want to appear tough.”) similarly occur in daily life (Gross, 2015).

### Emotion Regulation Strategies and Their Neural Representation

The number of regulation strategies, on the other hand, surely exceeds the diversity of goals. The various possible strategies can be differentiated by the point at which they intervene in the emotion generation process: Situation selection, situation modification, attentional deployment, cognitive change (also called cognitive reappraisal), and response modulation, each further characterized by different tactics and forms (Gross, 1998; Powers and LaBar, 2019). For instance, situation modification might be achieved by problem-solving, attentional deployment by distraction or selective attention, cognitive change by cognitive distancing “*from*” or reinterpretation “*of*” the situation, and response modulation by expression or suppression of the emotional display (also called expressive suppression). Therefore, common (cognitive) processes involved in the implementation of emotion regulation across strategies include attention and perspective-taking, self-control, and inhibition, goal updating and conflict monitoring, working memory, awareness of bodily states, and valuation (Ochsner and Gross, 2005; Ochsner et al., 2012; Gross, 2015). This is mirrored in the common neural activation patterns during emotion regulation. Emotional up- and down-regulation attempts are associated with the activation of cortical regions, including both the medial and lateral parts of the dorsal and ventral prefrontal cortex, the cingulate cortex, and the inferior parietal cortex, that influence or control subcortical, emotion-generating regions. These subcortical regions include the amygdala and (posterior) insula (Buhle et al., 2014; Frank et al., 2014; Kohn et al., 2014; Morawetz et al., 2017a, b; Berboth and Morawetz, 2021). Further, it has been shown that cortical regions are selectively recruited for the regulation of positive against negative stimuli (Golkar et al., 2012), for different regulation goals such as up- against down-regulation (Ochsner et al., 2002; Morawetz et al., 2016, 2017a), and for different regulation tactics, e.g., cognitive distancing, reinterpretation, expressive suppression, or distraction (Kanske et al., 2011; Dörfel et al., 2014; Morawetz et al., 2017b; Langner et al., 2018). These findings point to different context-dependent, flexibly changing patterns of co-activation of brain structures accompanying the common and potentially indispensable control network of emotion regulation.

### Flexible Regulation of Emotions and Psychopathology

Although the theoretical foundation of the emotion regulation construct highlighted the dynamic interplay between persons and situations (Gross and Jazaieri, 2014), much of the research in this area has adopted a relatively static approach that categorizes single regulatory strategies as inherently adaptive (e.g., reappraisal and expression) or inherently maladaptive (e.g., avoidance and expressive suppression). This view is backed by several research findings. For instance, the meta-analysis by Aldao et al. (2010) found the dispositional use of rumination, avoidance, and expressive suppression positively and strongly related to psychopathology, whereas the use of problem-solving, reappraisal, and acceptance negatively (albeit not as strongly) associated with psychopathology. Similarly, expressive suppression has been negatively linked to different characteristics of mental health, whereas reappraisal positively predicted mental health, suggesting a common characteristic across different diagnostic categories (Hu et al., 2014).

No doubt, emotion dysregulation in general represents a symptom across several psychiatric disorders (Gross and Jazaieri, 2014). This is paralleled by brain imaging studies in patients with various psychiatric disorders, also pointing to dysregulation in neural emotion regulation networks (Taylor and Liberzon, 2007; Gaebler et al., 2014; Rabinak et al., 2014; Wackerhagen et al., 2017, 2018; Fitzgerald et al., 2019; Khodadadifar et al., 2022; Poon et al., 2022).

However, in the last decade, the distinction between merely adaptive and maladaptive emotion regulation strategies has been challenged (see for instance, Sheppes and Gross, 2011; Aldao, 2013; Troy et al., 2013; Kashdan et al., 2015). A growing body of research has demonstrated that the efficacy of specific strategies varies markedly across situations and individuals (Bonanno et al., 2011; Sheppes et al., 2014; Birk and Bonanno, 2016; Troy et al., 2017). For instance, Troy et al. (2013) showed initial evidence that reappraisal is adaptive when stressors are uncontrollable but maladaptive when the situation can be controlled. Aldao and Nolen-Hoeksema (2012a) reported that when dispositional use of maladaptive strategies was low, the use of adaptive strategies was unrelated to psychopathology. In contrast, at high levels of maladaptive strategy use, adaptive strategies were negatively related to psychopathology. Additionally, Aldao and Nolen-Hoeksema (2012b) showed that not the mere use of adaptive strategies, but the variability in the implementation of acceptance and problem solving predicted lower levels of psychopathology.

In line with this, a growing number of studies report findings about the repertoire of emotion regulation strategies and their relationship to psychopathology, personality disorders, and personality traits (Lougheed and Hollenstein, 2012; Dixon-Gordon et al., 2015). The term “repertoire” can be defined as the ability to utilize a wide range of regulatory strategies in divergent contextual demands and opportunities (Bonanno and Burton, 2013, p. 594), fostering regulatory flexibility. Similarly, several other authors highlight the importance of flexibility in strategy use and assume that the regulatory process ideally results in an optimal level of emotion dynamics in order to produce appropriate responses and therefore a healthy adaptation to the demands of the environment (Kashdan and Rottenberg, 2010; Aldao, 2013; Aldao et al., 2015).

Additionally, Pruessner et al. (2020) point to the importance of individual differences in cognitive control for emotion regulation flexibility. Thus, it could also be assumed that personality traits rather influence regulatory flexibility than the frequency of using single emotion regulation strategies (see Scheffel et al., 2019; Dörfel et al., 2020). For instance, neuroticism has been shown to be negatively related to general psychological flexibility, while conscientiousness was positively associated with psychological flexibility (Latzman and Masuda, 2013).

Nonetheless, the flexible and adaptive choice from a repertoire of regulation strategies and regulation tactics and its interaction with personality dispositions has been scarcely investigated so far (Kobylinska and Kusev, 2019). Moreover, compelling evidence for the influence of different psychopathological, emotional, resilience, and personality traits on expressive flexibility is still lacking.

### Flexible Regulation of Emotional Expression

Flexibility not only refers to the use of regulatory strategies from different categories (e.g., cognitive change vs. response modulation). Recently, there have been investigations into the flexible use of different tactics and forms from one strategy category, for instance for cognitive reappraisal (Weber et al., 2014), as well as for emotional expression and suppression (Bonanno et al., 2004; Chen et al., 2018).

Flexibility in emotional expressive regulation, or expressive flexibility (EF), has been studied using a within-subjects laboratory paradigm, to investigate the participants’ ability to both up- and downregulate (enhance and suppress, respectively) displayed emotions (Bonanno et al., 2004; Westphal et al., 2010; Gupta and Bonanno, 2011). In this context, expressive enhancement was defined as a person’s ability to intentionally modify their emotional display to be more expressive relative to their own baseline level of expressiveness, from which an observer could more easily guess what the person was feeling; while emotional suppression was defined as a person’s ability to be less expressive than their own baseline from which an observer could not easily guess what the person was feeling (Bonanno et al., 2004, pp. 483–484). Accordingly, Burton and Bonanno (2016) recently developed a self-report measure of EF, the Flexible Regulation of Emotional Expression (FREE) Scale. For instance, it evaluates to what extent participants would be able to enhance or suppress their emotional expression compared to how they were actually feeling in a hypothetical social situation. Burton and Bonanno (2016) could prove a hierarchical factor structure of the FREE scale containing four factors or sub-scales that load into two factors of higher order: (i) enhancing positive emotion (show a more positive emotional expression e.g., in the following situation “A friend wins an award for a sport that doesn’t interest you.”); (ii) enhancing negative emotion (show a more negative emotional expression e.g., when “Your friend is telling you about what a terrible day they had.”); (iii) suppressing positive emotion (conceal a positive emotion or decrease a positive emotional expression e.g., when “You are in a training session and you see an accidentally funny typo in the presenter’s slideshow.”); and (iv) suppressing negative emotion (conceal a negative emotion or decrease a negative emotional expression e.g., “After you have a very irritating and stressful day, a sometimes annoying neighbor stops by to say hello.”). These abilities of expressive enhancement and expressive suppression correlate with experimental measures of these same abilities and have demonstrated similar relationships to measures of emotion regulation, and personality (Burton and Bonanno, 2016).

A previous validation of the FREE to the Chinese population showed that suppression ability was associated with fewer symptoms of depression and anxiety while, in keeping with the flexibility concept, the interaction of expressive and suppressive abilities predicted higher life satisfaction (Chen et al., 2018). However, it remains unclear: (i) whether the FREE scale may serve as a valid instrument to simultaneously assess the ability to enhance and suppress emotional expressions; and (ii) whether the association between affective symptoms and emotional flexibility can be generalized to further populations. Accordingly, we first translated the FREE-scale for use in the German population. We provide a comprehensive validation of its internal reliability and its construct validity *via* confirmatory factor analysis. We expected a hierarchical factor structure with four subscales that collapse into two factors as previously suggested (Burton and Bonanno, 2016; Chen et al., 2018). Finally, according to our hypothesis, we evaluated whether enhancement and suppression abilities, as well as overall expressive flexibility are associated with psychopathological symptoms (i.e., depression, anxiety, stress, and overall mental health), emotion regulation skills, coping strategies, and personality traits.

## Methods

**Figure 1:**
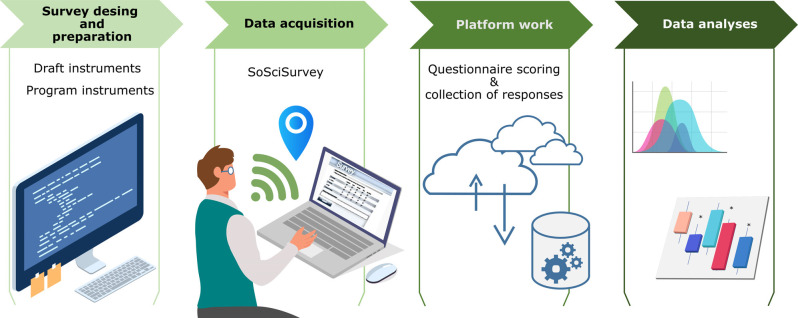
Schematic presentation of the experimental design. From left to right: After designing the online questionnaires (directly from article questionnaires), they were put online and recruitment of participants started. The questionnaires were automatically scored and collected to a local computer for subsequent statistical analyses.

“We report how we determined our sample size, all data exclusions (if any), all manipulations, and all measures in the study” (Simmons et al., 2012). A visual overview of the experimental design is shown in Figure 1.

### Participants and Procedure

The study consists of two waves of an online survey in spring 2018 (*N* = 275) and winter 2018/19 (*N* = 76). Participants of the second wave also underwent a 6 min resting HRV (heart rate variability) measurement in the psychophysiological laboratory. In wave two, we also added an assessment on psychotherapeutic drug intake as exclusion and German mother tongue as inclusion criteria. The sample size was determined by the duration of each wave of the survey. There was a time limit because the surveys were part of three Bachelor theses. Due to these time constraints in sample size determination, no power analysis was conducted. A sensitivity analysis revealed an effect size of |ρ| = 0.22 that could be detected by our study at a power of 0.95 and an effect size of |ρ| = 0.16 at a power of 0.80 (Faul et al., 2009). Additionally, according to Schönbrodt and Perugini (2013), the sample size is sufficient to detect effect sizes of *ρ* > 0.1 (+/– 0.15) with a confidence level of 95%.

Three-hundred and fifty-one participants located in Germany (verified *via* postal codes) were recruited through an online platform for subject recruitment at a major university in Germany based in ORSEE (Greiner, 2004), by means of advertisements in social media and *via* flyers at two major universities in Germany. The sample represents a convenience sample, no snowball sampling technique was applied. Participants were invited to an online survey created with SoSciSurvey (Leiner, 2014), an online survey tool that allows researchers to upload and distribute surveys to a pool of participants who complete study procedures from their personal computers. From this initial cohort, 129 participants had to be excluded due to incomplete data (*N* = 113), repeated participation (*N* = 9), careless response behavior (*N* = 2), or due to their participation in the pre-test phase (*N* = 5). Finally, 222 participants (173 female, mean age ± SD = 23.36 ± 5.05 years; wave 1 *N* = 154, wave 2 *N* = 68) were included in this study.

Informed consent was obtained from the participants at the start of the online survey, the survey continued only after the participants actively agreed to proceed. The study protocol was approved by the institutional review board of the Technische Universität Dresden and conducted in accordance with the principles expressed in the Declaration of Helsinki.

### Flexibility in Emotional Expressive Regulation

We used the Flexible Regulation of Emotional Expression (FREE) Scale (Burton and Bonanno, 2016) to measure the ability to regulate emotional expression. First, all the 16 scenarios (items) were translated into German by two bilingual researchers, and revised several times (also by trained psychologists) to ensure the maximum similarity to the original scale (see Supplementary Material). For each item of the FREE scale participants indicated their ability to “be even more expressive than usual of how [they] were feeling” or to “conceal how [they] were feeling” on a 6-point scale. Herein, higher ratings corresponded to greater self-rated ability to modulate the expression of emotions (i.e., 0 = unable/not at all, 6 = very much).

The FREE scale measures four different emotion expression abilities, namely enhancing positive emotion (items 1–4), enhancing negative emotion (items 5–8), suppressing positive emotion (items 9–12), and suppressing negative emotion (items 13–16). Sum scores are calculated for each of the four subscales. The positive and negative enhancing subscales are subsequently combined, resulting in an “expressive enhancement” ability factor (Cronbach’s α = 0.83, for the present study). Similarly, the two positive and negative suppressing subscales are combined to derive a “suppress” regulation ability factor (Cronbach’s α = 0.71; Burton and Bonanno, 2016). A sum score is obtained by totaling both enhance and suppress factor scores and a polarity score by getting the absolute value of the difference between enhancement and suppression. The EF (Cronbach’s α = 0.81) is finally calculated by subtracting the polarity score from the sum score, where higher scores indicate greater flexibility in regulating emotional expressions.

### Assessment Of Psychopathological Symptoms and Mental Health

#### Psychopathology Symptoms

To assess the affective (i.e., psychopathological) state, the short version of the Depression, Anxiety, and Stress Scale (DASS) was used (Lovibond and Lovibond, 1995; Nilges and Essau, 2015). The DASS is a 21-item self-report screening instrument composed of three subscales for measuring each of the exposure to depression, anxiety, and tension/stress symptoms. A four-point severity/frequency scale (0 = not true/never to 3 = true/most of the time) is used to rate the extent to which the participants have experienced each symptom over the previous week. Higher ratings of each subscale indicate high or severe negative symptoms. Cronbach’s α in the present study was 0.9 for depression, 0.75 for anxiety, and 0.82 for stress/tension symptoms.

#### Well-Being

General mental well-being was measured using the Well-Being Index (Bech, 2004). This is a five-item questionnaire rated on a 6-point Likert scale (0 = not present to 5 = constantly present) which is translated into a single global score. Higher scores correspond to higher well-being. Cronbach’s α in the present study was 0.81.

#### Emotion Regulation

To measure emotion regulation skills, the 27-item version of the Emotion Regulation Skills Questionnaire (SEK-27, Berking and Znoj, 2008) was administered. Successful emotional regulation use is assessed through the following nine subscales with three items per skill: awareness, sensations, clarity, understanding, modification, acceptance, tolerance, readiness to confront distressing situations, and compassionate self-support. Each item is assessed on a 5-point Likert-type scale (0 = never to 4 = almost always). In addition to the subscales, a total score for successful emotion regulation can be computed as the average of all items. Cronbach’s α in the present study was 0.81 for awareness, 0.74 sensations, 0.81 for clarity, 0.78 for understanding, 0.83 for tolerance, 0.69 for acceptance, 0.78 for self-support, 0.78 for readiness to confront, 0.8 for modification, and 0.92 for total successful emotion regulation.

The Emotion Regulation Questionnaire (ERQ) is a 10-item self-report questionnaire designed to assess individual differences in the habitual use of emotion regulation strategies: cognitive reappraisal (six items) and expressive suppression (four items; Abler and Kessler, 2009). In our sample, Cronbach’s α was 0.87 for cognitive reappraisal and 0.77 for expressive suppression.

#### Coping Strategies

The German version of the resilience scale (RS-11, Schumacher et al., 2005) was used to measure participants’ coping strategies. This scale is designed as a measure to assess the ability to bounce back or recover from stress or when faced with a burdensome life event. Participants rated their accordance of eleven resilience items on a 7-point Likert scale ranging from 1 (never) to 7 (always). The internal consistency in this study was α = 0.83.

Further, coping behavior was measured using the self-report Coping Flexibility Scale (CFS, Kato, 2012). The CFS measures individuals’ perception of their own ability to implement flexible coping in situations in general. CSF is composed of two constructs, namely, evaluation coping (how well a person monitors and evaluates coping outcomes) and adaptive coping (how well a person uses an alternative coping strategy to produce a desirable outcome). The two subscales consist of five items each. Cronbach’s α for the sample in the present study was 0.47 for evaluation and 0.88 for adaptation. An undergraduate and a trained psychologist with a doctorate degree translated the English version into German and a native English speaker and psychology graduate provided a back-translation, which was evaluated by the trained psychologist with a doctorate degree.

To assess perceived self-efficacy the German version of the generalized self-efficacy scale (GSE) was used. The GSE is a 10-item psychometric scale that is designed to assess optimistic self-beliefs to cope with daily problems and adapt to stressful life events (Schwarzer and Jerusalem, 2003). The GSE is evaluated with a Likert scale ranging from 1 (not true at all) to 4 (exactly true). In contrast to other scales that assess optimism, the GSE explicitly refers to personal agency, i.e., the belief that one’s actions are responsible for successful outcomes (Schwarzer and Jerusalem, 1995, 2003). Cronbach’s α in the present study was 0.85.

#### Personality

The Big-Five-Inventory-10 (BFI-10) is a short 10-item scale measuring the Big Five personality traits extraversion, agreeableness, conscientiousness, emotional stability, and openness (Rammstedt and John, 2007). The scale was simultaneously developed in English and German and is designed for contexts in which respondents’ time is severely limited. BFI-10 ratings range from “strongly disagree” (1) to “strongly agree” (5). Cronbach’s α for BFI-10 in the present study was 0.66 for emotional stability, 0.81 for extraversion, 0.37 for agreeableness, 0.42 for conscientiousness, and 0.6 for openness.

#### Physical Complaints

To assess general physical complaints, we used the Giessen Complaint Questionnaire (GBB-24, Giessener Beschwerdebogen). The GBB-24 evaluates such physical complaints as exhaustion tendency, gastric trouble, rheumatic pains, and heart complaints in terms of whether they are fully or partly psychosomatically induced (Brahler and Scheer, 1979). Each question is ranked with a Likert scale ranging from 0 (not at all) to 4 (strongly) and the total score represents the overall subjective complaints. The higher the scores, the higher the exhaustion tendency. Cronbach’s α in the present study was 0.84.

Additional measurements not reported in this study were: Flexible emotion regulation, assessed through a newly developed self-report scale (FlexER, Dörfel et al., 2019), reappraisal inventiveness assessed with the reappraisal inventiveness test (RIT, Weber et al., 2014), and need for cognition (NFC, Bless et al., 1994).

### Statistical Analyses

#### Demographical Variables

First, given that the sample included more females, the demographic variables between males and females were compared using χ^2^ tests for categorical variables (i.e., education level, professional qualifications, and family status), and *T*-tests were conducted to evaluate continuous variables (i.e., age).

#### Validation of the German Version of the FREE Scale

Following, to evaluate the internal consistency and reliability of the FREE scale, the Cronbach’s α (Cronbach, 1951) was calculated across items, across subscales, and across scenarios as the mean of all possible split half reliabilities (corrected for test length) using the R package psych [v1.8.12, Revelle, 2017] and MATLAB (R2017b; The MathWorks, Inc., Natick, MA, USA). Cronbach’s α is a positive function of the number of items in the test as well as the average inter-correlation of the items in the test. It is calculated by comparing the shared item variances relative to the total test variance.

Based on our expectation about the model structure of the FREE-scale, we performed a confirmatory factor analysis (CFA) with the maximum likelihood (ML) function with Satorra-Bartlett correction (because of non-normal distribution of all FREE items) using the R package lavaan (latent variable analysis; v 0.6–3). As the basis to validate the hypothesized model structure of the FREE scale, we tested models of different complexity, including a “single factor” model consisting of all 16 items, an “expressive regulation ability” model consisting of dual latent factors (i.e., enhance and suppress each with eight items as loadings), an “emotional valence” model also consisting of dual factors (i.e., negative and positive emotionally valence each with eight items as loadings). Finally, based on the suggested interrelation between the positive and negative valences for enhancement and suppression abilities (Burton and Bonanno, 2016), a “correlated factor” model including all four subscales (four items each) was tested. Several indicators of the model fit of the CFA were calculated, such as the overall fit and discrepancy of the model with chi-square (*χ*^2^) the root mean square error of approximation (RMSEA), the Standardized Root Mean Square Residual (SRMR), the Bentler comparative fit index (CFI), and Tucker Lewis Index (TLI), Akaike Information Criteria (AIC), and the Bayesian Information Criteria (BIC). Following suggestions by Hu and Bentler (1999), acceptable model fit was defined by the following criteria: RMSEA ≤ 0.06, SRMR < 0.08, CFI ≥ 0.95, and TLI ≥ 0.95. CFA models were compared against the “correlated factor” model using the likelihood ratio test (LRT) with the function “lavTest LRT” (wrapped through the “ANOVA” function) based on the approximation described in Satorra and Bentler (2001).

#### Association Between Flexibility in Emotional Expressive Regulation With Indicators of Psychopathology Symptoms and Mental Health

The task of determining which predictors are associated with a given response is not a simple task. When selecting the instruments for a linear model, looking at individual *p*-values is common. However, this could be misleading. For instance, if the instruments are highly correlated the *p*-values will also be high leading to the incorrect inference that those instruments are not important predictors. On the other hand, irrelevant instruments that are not associated with the response may be included in further analyses, adding unnecessary complexity to the model. Therefore, algorithms that automatically reduce the number of predictors, which in turn improves model interpretability, are preferred. In this line, the Least Absolute Shrinkage and Selection Operator (LASSO, Tibshirani, 2011), a penalized least-squares technique, possesses the ability to predictor selection and shrinkage in reasonable running time. Here, we used 10-fold cross-validation (James et al., 2013) in order to determine which set of predictors was better on each particular response, including the ability to enhance emotional and suppress emotional expression as well as overall expressive flexibility. The LASSO solves the penalized shrinkage problem of the *l*1 norm of β on the form:


$$l_{\lambda }^{L}\left(\beta \right)=\underset{\beta_{0},\beta }{\min}\left\{\frac{1}{2N}\sum_{i=1}^{N}{\left(y_{1}-\beta_{0}-x_{i}^{T}\beta \right)}^{2}+\lambda \sum_{j=1}^{p}\left|\beta_{j}\right|\right\}$$

Where N is the number of observations, *y_i_* is the response at observation *i*, *x_i_* is a vector of predictors of length *p* at observation *i*. The parameters *β*_0_ and *β* are a scalar and a vector of length *p*, respectively. λ is a nonnegative regularization parameter corresponding to one value of Lambda, such that as λ increases, the number of nonzero components of *β* decreases. *β* coefficient estimates forced to be exactly equal to zero are discarded from the model.

Given that the LASSO does not provide information about the specific relationship between the predictors and the responses, the rank partial correlation coefficients were used to ascertain if enhance or suppress regulation abilities and EF acted as independent determinants of psychopathological symptoms as well as personality, well-being, and coping strategies. All models included gender and age as covariates. Correction for multiple comparisons was performed across measuring instruments as well as domains of the FREE scale using false discovery rate (FDR) at 95% confidence.

As the flexibility concept proposes that enhance and suppress measures are inter-related to affective symptoms, we further fitted a GLM model to determine how well the interaction term between expressive suppress or enhancement abilities increases the relationship to psychopathological symptoms (i.e., depression, stress, and anxiety), while correcting the effects of age and gender. Resulting in a model of the form:


$$\hat{R}=\beta_{0}+\beta_{1}Age+\beta_{2}Gender+\beta_{3}\left(E\times S\right)$$

Where $\hat{R}$ is the response variable of interest (i.e., depression, stress, or anxiety symptoms), β_0_ is a constant term, *β*_1_ Age + *β*_2_ Gender represents the additive effects of the covariates Age and Gender, and *β*_3_ (*E* × *S*) is the interaction term between enhance and suppress expression abilities. All GLM analyses were conducted on MATLAB. When examining interaction effects, the main effect of one predictor (*E*) depends on the specific value of the second predictor (*S*) in the fitted regression function. This is known as a conditional effect. Thus, in our study, each interaction model evaluates the main effects, as well as the conditional effects of the minimum and maximum values of the response instruments as well as the average value of the minimum and maximum.

## Results

### Participants

Of the final 222 included participants, 94.1% (the majority) of participants had completed a minimum of 12 years of education at the time of their participation (equivalent to German Abitur), whereas the remaining 5.9% had undergone approximately 10 years of education (German MittlereReife or Hauptschule). 65.8% of the participants had not completed their professional qualification, yet, while the others had completed vocational training (12.6%), bachelor’s (14.4%), master’s (5.4%), or Ph.D. (1.8%). Additionally, 45% of the participants stated that they currently were in a relationship or married and 94.6% reported that they had no children. Despite the majority of participants (77.9%) being female, no differences existed related to gender for age (*P* = 0.396, *T* = 0.85), level of education (*P* = 0.44, χ^2^ = 0.61), nor professional qualification (*P* = 0.62, χ^2^ = 2.64). Sex differences were found solely for family status (*P* = 0.01; χ^2^ = 6.4), where within females there was a similar number of single and non-single participants, whereas males had a higher proportion of single participants.

### Evaluation and Validation of the FREE Scale (German Version)

The reliability analysis showed that the internal consistencies (Cronbach’s α) of the four subscales were acceptable: namely, enhance positive emotion (α = 0.73), enhance negative emotion (α = 0.73), suppress positive emotion (α = 0.69), and suppress negative emotion (α = 0.60). Whereas for the two second-order factors (eight-item composites), namely enhancement (α = 0.83) and suppression (α = 0.71) abilities, the reliability estimates ranged from good to acceptable (Table 1). Overall, when considering all of the individual items, Cronbach’s α indicated good reliability of the FREE scale for emotional expression flexibility (α = 0.81; Table 1).

**Table 1:** Reliability and internal consistency.

**Scale**	**Measure**	**Cronbach’s α**
FREE	expressive enhancement	0.83
	suppress regulation	0.71
	EF	0.81
DASS	depression	0.90
	anxiety	0.75
	stress/tension	0.82
Well-being index		0.81
SEK-27	sensations	0.74
	clarity	0.81
	understanding	0.78
	tolerance	0.83
	acceptance	0.69
	self-support	0.78
	readiness to confront	0.78
	modification	0.80
	total successful emotion regulation	0.92
ERQ	cognitive reappraisal	0.87
	expressive suppression	0.77
RS-11		0.83
CFS	evaluation	0.47
	adaptation	0.88
GSE		0.85
BFI-10	emotional stability	0.66
	extraversion	0.81
	agreeableness	0.37
	conscientiousness	0.42
	openness	0.60
GBB-24		0.84

*Assessment of the correlation between multiple items in particular tests that are intended to measure the same construct. FREE, Flexible Regulation of Emotional Expression; DASS, Depression, Anxiety, and Stress Scale; SEK-27, Emotion Regulation Skills Questionnaire; ERQ, Emotion Regulation Questionnaire; RS-11, Resilience scale; CFS, Coping Flexibility Scale; GSE, Generalized Self-Efficacy scale; BFI-10, Big-Five-Inventory-10; GBB-24, Giessen Complaint Questionnaire*.

**Figure 2:**
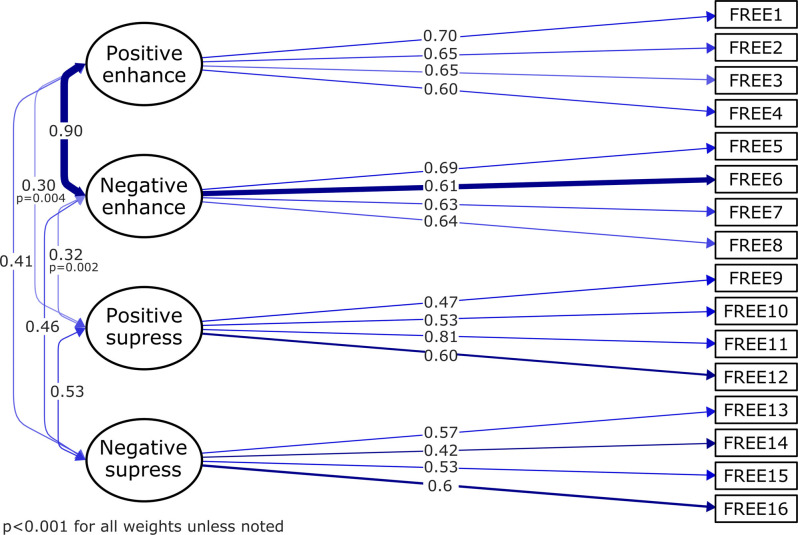
Path diagram of the confirmatory factor analysis (CFA) “correlated factor” model. This figure shows the standardized factor loadings for the “correlated factor” model of the FREE scale. The rectangles indicate the 16 items of the scale; the four directly connected ellipses represent the latent variables (subscales). The lines represent the causal effects from the first-order factors to the individual items. The line thickness/continuity indicates the magnitude of the loading factors.

As expected based on previous research (Burton and Bonanno, 2016; Chen et al., 2018), the “single factor” model didnot fit the data well (χ^2^_(104)corr_ = 305.407, *P* < 0.001, RMSEA_corr_ = 0.093, SRMR = 0.103, CFI_corr_ = 0.71, TLI_corr_ = 0.67, AIC = 10,757.830; BIC = 10,866.716). This was the same case for both dual-factor models, “expressive regulation ability” (factors: enhancement and supression; χ^2^_(103)corr_ = 181.743, *P* < 0.001, RMSEA_corr_ = 0.059, SRMR = 0.074, CFI_corr_ = 0.888, and TLI_corr_ = 0.869, AIC = 10,613.223; BIC = 10,725.511) and “emotional valence” (factors: positive and negative; χ^2^_(103)corr_ = 301.884, *P* < 0.001, RMSEA_corr_ = 0.093, SRMR = 0.103, CFI_corr_ = 0.716, TLI_corr_ = 0.669, AIC = 10,755.400; BIC = 10,867.688) models. The fit indices suggested that the “correlated factor” model had an adequate but not entirely acceptable fit to the data (χ^2^_(98)corr_ = 133.962, *P* = 0.009; RMSEA_corr_ = 0.041, SRMR = 0.060, CFI_corr_ = 0.949, TLI_corr_ = 0.937, AIC = 10,566.919, and BIC = 10,696.22). Figure 2 depicts the complete specification of the “correlated factor” (i.e., four-factor, sub-scales) model. The latent factors were permitted to covariate based on prior evidence of a relationship between these dimensions.

When testing the “correlated factor”model against the rest of models, the “correlated factor” model fitted the data significantly better than a model with a single latent factor, χ^2^_(6)_ = 202.91, *P* < 0.001, the “expressive regulation ability” model (i.e., enhance and suppress latent factors; χ^2^_(5)_ = 56.304, *P* < 0.001), and the “emotional valence” model (i.e., negative and positive emotionally valence latent factors; χ^2^_(5)_ = 198.304, *P* < 0.001). Therefore, the CFA with a correlated factor structure was the best model.

In the “correlated factor” model, the high standardized parameter estimates between positive and negative enhancement (=0.90) evidenced that these two latent variables are strongly interrelated, in other words, the two converge to load on the same factor and likely represent the same construct, i.e., expressive enhancement. This indicates that they similarly measure the ability for emotional expressive enhancement, while the interrelation between negative and positive suppression factors was less convergent (=0.53). Thus, even if these second order factors could not be completely confirmed by CFA, we decided to keep the originally proposed scale structure of the English version of the FREE for our next analyses. On the contrary, the fact that relatively low standardized parameter estimates (range 0.30–0.46) were found between the two suppression and the two enhancement factors, depicts good discriminant validity, or low convergence on separate factors.

**Figure 3:**
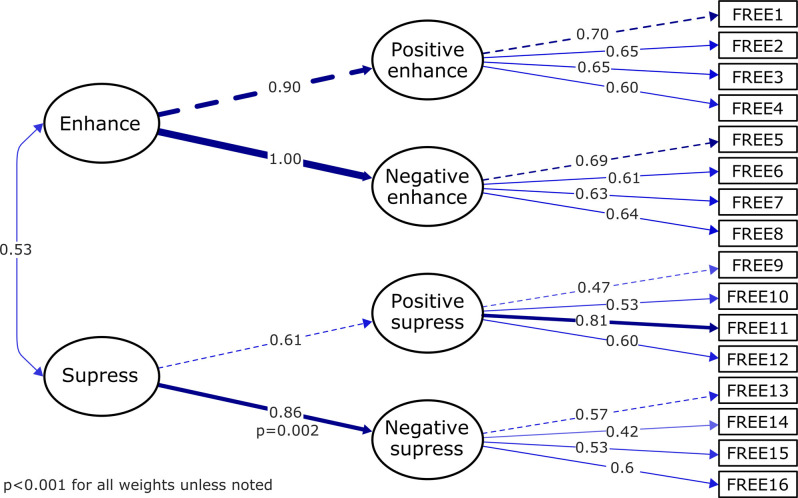
Path diagram of the confirmatory factor analysis (CFA) “hierarchical factor” model. The rectangles indicate the 16 items of the scale, the four directly connected ellipses represent the latent variables, and the two further ellipses are the second-order factors that load into the overall expressive flexibility ability. The lines represent the causal effects of second-order factor to the first-order factors and from those to the individual items, color by the corresponding standardized factor loading also written as a number. The line thickness/continuity indicates the magnitude of the loading factors.

Despite being tested, we decided against including a highly complex model with four first order factors (positive enhance, negative enhance, positive suppress, negative suppress; four items each) and two second order factors (enhancement, suppression ability). This was because the final medium sample size could lead to out of range standardized parameter estimates (i.e., values equal to or greater than 1) on the subscales, related to the expected high relationship among them (see Figure 3 and Table 2 for details on this model).

**Table 2:** Confirmatory factor analyses (CFA) implemented to verify the factor structure of the Flexible Regulation of Emotional Expression (FREE) scale. CFA was used further served to test the hypothesis that a relationship between FREE variables and their underlying latent constructs exists.

**Model**	**FREE Subscale**	**chi-square (χ^2^)**	***p*-value**	**RMSEA**	**SRMR**	**CFI**	**TLI**	**AIC**	**BIC**
Single factor model		305.407	<0.001	0.093	0.103	0.71	0.67	10,757.83	10,866.72
Two factor models	Expressive regulation^†^	181.743	<0.001	0.059	0.074	0.888	0.869	10,613.22	10,725.51
	Emotional valence^¶^	301.884	<0.001	0.093	0.103	0.716	0.669	10,755.4	10,867.69
Correlated factor model	enhance positive emotion	133.962	0.009	0.041	0.06	0.949	0.937	10,566.92	10,696.22
	enhance negative emotion								
	suppress positive emotion								
	suppress negative emotion								
Hierarchical model*	positive enhance (enhancement)	165.849	<0.001	0.055	0.06	0.92	0.903	10,564.47	10,690.83
	negative enhance (enhancement)								
	positive suppress (suppression)								
	negative suppress (enhancement)								

RMSEA, Root Mean Square Error of Approximation; SRMR, Standardized Root Mean Square Residual; CFI, Bentler Comparative Fit Index; TLI, Tucker-Lewis Index; AIC, Akaike Information Criteria; BIC, Bayesian Information Criteria. ^†^Expressive regulation model factors: enhancement and suppression abilities. ^¶^Emotional valence model factors: negative and positive regulation. *The hierarchical model consists of four first order factors (positive enhance, negative enhance, positive suppress, negative suppress, and two second order factors (enhancement, suppression abilities).

### Association Between Flexibility in Emotional Expressive Regulation With Indicators of Psychopathology Symptoms and Mental Health

It can be expected that expressive enhancement and suppression abilities, as well as the overall flexibility, do not associate to the same degree with the rest of the study instruments measuring psychopathological symptoms (i.e., depression, stress, anxiety, and mental well-being), emotion regulation skills, coping strategies, and personality traits. Accordingly, we first opted for a data mining approach in which by means of a LASSO regression we objectively disregarded instruments that do not show any association. Of the initial 26 instruments (see assessed instruments in the “Methods” section), the LASSO selected 21 for enhance and suppress abilities, as well as for the EF. For all three factors, LASSO disregarded most of the personality traits from the BFI-including extraversion, openness, conscientiousness, and agreeableness, and the subscale “understanding” from the SEK-27 (Table 3).

**Table 3:** LASSO coefficients of each of the Second-Order Factors and Overall Expressive Flexibility (EF) of the FREE Scale.

	**LASSO coefficients**		
	**FREE-Enhance**	**FREE-Suppress**	**FREE-Overall Flexibility**
RES11-Resilience	0.316	0.238	0.068
GBB24-Physical complaints	0.213	0.117	0.041
WHO5-Well-Being	0.028	−0.102	−0.008
DASS-Depression	−0.118	0.089	−0.012
DASS-Anxiety	−0.075	−0.095	−0.057
DASS-Stress	0.220	−0.112	0.023
BFI-Emotional Stability	6.336	−3.919	0.862
BFI-Extraversion	0	0	0
BFI-Openness	0	0	0
BFI-Conscientiousness	0	0	0
BFI-Agreeableness	0	0	0
CFS-Evaluation	0.018	0.006	0.002
CFS-Adaptation	0.159	0.185	0.035
SEK27-Awareness	7.532	−3.506	0.943
SEK27-Clarity	5.703	−2.437	1.034
SEK27-Sensations	6.332	−3.452	0.830
SEK27-Understanding	0	0	0
SEK27-Acceptance	6.474	−1.207	1.205
SEK27-Tolerance	4.945	−4.909	0.543
SEK27-Self-Support	4.864	−4.088	0.702
SEK27-Readiness to Confront	5.240	−2.914	0.913
SEK27-Modification	5.260	−3.833	0.680
SEK27-Total	−1.964	1.210	−0.279
ERQ-Reappraisal	0.913	0.490	0.256
ERQ-Suppression	0.437	1.414	0.187
GSE-Self-Efficacy	0.075	0.032	−0.003

*Instruments with LASSO coefficients equal to zero are disregarded from subsequent analyses*.

Table 4 depicts the results of the partial rank correlation analyses on the 21 remaining instruments output from the LASSO but colored according to the direction of the partial correlation coefficients. Regarding the expressive suppressionand enhancement abilities different scenarios emerged. The ability for expressive suppression showed negative associations with DASS-depression (*r* = −0.19, *P*_FDR_ = 0.009), and DASS-stress (*r* = −0.16, *P*_FDR_ = 0.025), while no association was seen between expressive suppression abilities and DASS-anxiety (*r* = −0.06, *P*_FDR_ > 0.05). Enhancement abilities showed no association with any DASS affective symptoms after correction for multiple comparisons. For measures of emotion regulation skills (SEK), expressive suppression but not enhancement was associated with higher readiness to confront distressing situations (*r* = 0.25, *P*_FDR_ = 0.001), compassionate self-support (*r* = 0.2, *P*_FDR_ = 0.008), and tolerance (*r* = 0.19, *P*_FDR_ = 0.01). For the remaining instruments, expressive suppression abilities showed stronger associations, except for BFI-Emotional stability (*r* = 0.24, *P*_FDR_ = 0.002), SEK27-Awareness (*r* = 0.23, *P*_FDR_ = 0.004), SEK27-Clarity (*r* = 0.22, *P*_FDR_ = 0.005), and SEK27-Sensations (*r* = 0.18, *P*_FDR_ = 0.022), where expressive enhancement showed stronger associations. Both, enhancement and suppression abilities showed moderate and highly significant associations with resilience (*r* = 0.32, *P*_FDR_ < 0.001), representing a coping marker.

**Table 4:** Rank correlations (including 95% confidence intervals) of suppress and enhance abilities and overall expressive flexibility (ef) with psychopathology, coping strategies, emotion regulation, well-being, and physical complaints.

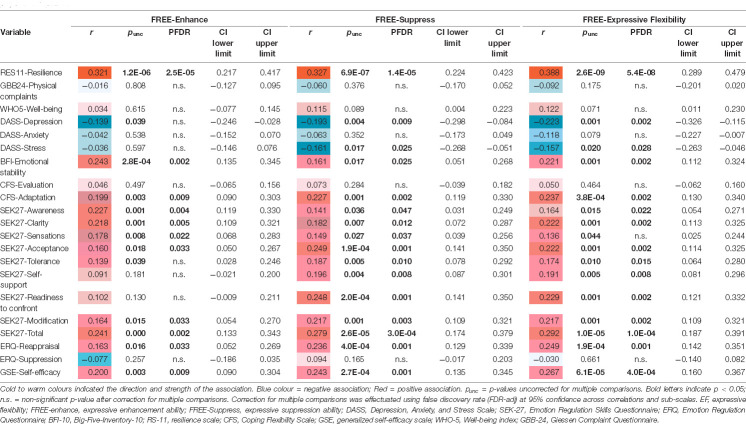

We further explored the association between EF and the rest of included instruments of psychological well-being (Table 4). Here, similarly to suppress and enhance abilities, the resilience score had the strongest positive association (*r* = 0.39, *P*_FDR_ = 5.4E-08), followed by SEK27-total (*r* = 0.29, *P*_FDR_ = 0.0001). Moreover, the strongest negative association was found with DASS-Depression (*r* = −0.22, *P*_FDR_ = 0.002), followed by DASS-stress (*r* = −0.16, *P*_FDR_ = 0.028).

The EF enhance and suppress abilities showed no associations with physical complaints (GBB-24), well-being (WHO-5), DASS-Anxiety, coping evaluation (CFS), SEK27-Sensations, nor emotional regulation suppression (ERQ; all *P*_FDR_ > 0.05).

**Figure 4:**
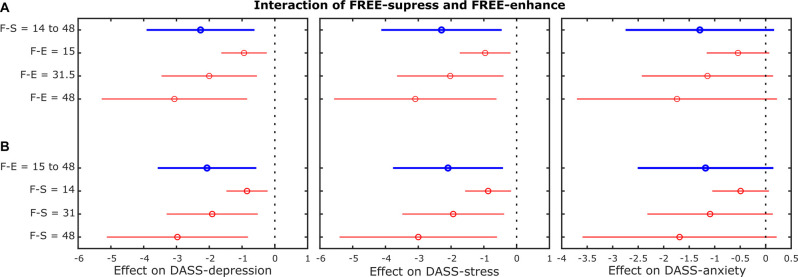
Interaction effects of suppress and enhance abilities on psychopathological symptoms. Linear regression model depicting the impact of a change in enhance abilities given the suppress abilities **(A)** and a change in suppress abilities given the enhance abilities **(B)** to predict depression (left), stress (middle), and anxiety (right) symptoms. The blue circles depict the main effects (i.e., the magnitude of the effect) and the red circles depict conditional effects (e.g., the impact of a change in enhance abilities for fixed values of suppress abilities) at the minimum value of each response instrument (upper), the maximum value of each response instrument (lower), and the average value of the minimum and maximum (middle). Blue and red lines show the upper and lower limits for the main effect at 95% confidence bounds for the effect values. The vertical black dashed line indicates the limit between decrease (negative) and increase (positive) effects. F-S, FREE Suppression; F-E, FREE Enhancement; DASS, Depression, Anxiety, and Stress Scale.

Beyond the association between expressive suppression and enhancement abilities with psychopathological symptoms (i.e., depressionand distress), and according to the hypothesized interrelation between expressive flexibility abilities and affective symptoms, the interaction models revealed that expressive suppression and enhancement abilities had an interactive effect (Figure 4) on DASS-depression (R-square = 0.037, Model-*F*_(1, 218)_ = 2.78, Model-*P* = 0.04; interaction-*T* = 2.75, interaction-*P* = 0.0066) and DASS-stress (R-square = 0.082, *F*_(1, 218)_ = 6.5, *p* = 0.0003; interaction-*T* = 2.47, interaction-*P* = 0.014), but not on DASS-anxiety (R-square = 0.025, *F*_220_ = 1, *P* = 0.14; interaction-*T* = 1.75, interaction-*P* = 0.08).

## Discussion

In the current study, we provide the German translation of the Flexible Regulation of Emotional Expression scale as the conceptual framework for testing whether expressive enhancement and suppression abilities are differentially associated with psychopathological symptoms as well as with personality traits, emotion regulation skills, and measures of coping strategies. The FREE scale was validated in a sample of young adults displaying adequate to good internal reliability, construct, and criterion validity. Evidencing its potential as a short, reliable, and valid instrument allows the simultaneous assessment of the ability to enhance and suppress emotional expressions as well as overall expressive flexibility.

The current findings are consistent with previous research (Chen et al., 2018) as both the total EF score and the two expressive suppression and enhancement abilities of the FREE show acceptable to good internal reliability. Consistent with the original report of the English version of the FREE-scale (Burton and Bonanno, 2016), the conducted CFA depicted high convergence for expressive enhance abilities, but this was not the case for the expressive suppression subscale. Thus, we did not validate the previously proposed hierarchical model structure with second-order factors. This suggests that although separate assessment of enhance and suppress dimensions is meaningful, it may vary from population to population.

Emotion regulation has been defined as a range of processes by which individuals reduce the onset, course, or experience of their emotional experiences, expressions, and physiology (Gross, 2015). However, regulation of emotions not only focuses on ameliorating negative effects, but also targets the maintenance, increase, or decrease of negative as well as positive emotions (Bonanno and Burton, 2013; Brans et al., 2013). Therefore, multiple routes and sources of emotional regulation exist, all of which modify at least one aspect of emotion such as physiology, attention, appraisals, experience, and expression (Thompson, 2011; Gross, 2014; Burton and Bonanno, 2016).

Altogether, personality traits (i.e., emotional stability), emotion regulation skills (i.e., awareness, acceptance, clarity, and modification), as well as coping strategies (i.e., resilience, adaptation, and the habitual use of reappraisal) positively correlated with expressive enhancement and suppression abilities and the overall EF. This not only provides evidence for the validity of the FREE-scale in a German population but also highlights the important role of emotional expressive abilities for mental health preservation, potentially acting as an underlying tool for the individual’s ability to adapt one’s level of control upwards or downwards as circumstances dictate. This idea is further corroborated by the negative association between higher emotional flexibility and suppression abilities with validated measures of psychopathological symptoms (i.e., depression and stress). These associations were enhanced when modeling the interaction between suppress and enhance ability, indicating that people with high flexibility abilities are psychologically healthier (i.e., have less psychopathological symptoms) while people with low flexibility are prone to present higher affective problems. In this context, it can be proposed that the ability to effectively and flexibly deal with emotions, in accordance with internal and external contextual demands, is fundamental to psychological health. This is in accordance with previous proposals on the positive relationship between coping strategies and psychological adjustment (Cheng et al., 2014), where higher levels of flexibility would predict fewer psychological symptoms (Waugh et al., 2011; Southward and Cheavens, 2017). Additionally, previous reports exist about the importance of emotion regulation for cognition in humans not only on a daily basis but at different life stages. For instance, research in infants has shown that early life stress has a meaningful and detrimental influence on prefrontal-subcortical networks and regulatory ability, as well as cognition (Gee et al., 2013; Arnsten, 2015), whereas adolescence is a key period, where individuals are more sensitive to reward and threat prompts but less able than adults to effectively recruit executive and control networks (Casey and Jones, 2010). Finally, at later life stages, mental health largely depends on the cognitive and brain reserve that has been accumulated across the years (Gonzalez-Escamilla et al., 2018) and may influence the levels of emotional stability and emotion regulation strategies at advanced ages (Carstensen et al., 2011). See Helion et al. (2019) for a throughout overview of this topic. What seems to be clear is that emotion regulation strategies are implicated in cognitive capabilities and impact brain functioning (Xiu et al., 2018; Moodie et al., 2020), and thus on health outcomes at different life stages. Unfortunately, across these different life stages, the relation between neural mechanisms (on a regional and networks level) and their influence on flexible emotion regulation abilities, particularly suppression, remain still largely elusive.

Emotional flexibility then helps to explain how dealing with stressful events and distress allows individuals to develop successful response approaches, which goes beyond generating positive emotions extending to the ability to expressively enhance and importantly to expressively suppress emotions. Hence, adding importance to the ability to inhibit emotional reactions. Moreover, the ability to flexibly regulate one’s emotions is key for dynamic and adaptive functioning across the life span. In this regard, according to our results and as previously discussed, a decay in the suppress abilities may be a risk factor or increase the risk for stress-related disorders (Visted et al., 2018; Coifman and Summers, 2019). Therefore, and as also evidenced in the current study, it is expected that suppress abilities are more related to psychopathological symptoms than enhance abilities (Chen et al., 2018). This accentuates the possibility that in mood and stress-related disorders emotional difficulties may result from the inflexible use of regulation strategies as the emotional distress exceeds the individual’s capacity to favorably implement an appropriate strategy. Of notice, even if an interaction between suppress and enhancement abilities for anxiety symptoms was found, individual associations with anxiety were not attested, and, thus, no further inference can be made in this direction. This is summed to the lack of evidence on the interrelations between supress abilities, affective disorders or psychopathology symptoms, and cognitive involvement and their dependence on the neural circuitry.

### Limitations and Further Directions

Limitations of our study are related to, first, the sample and second the cross-sectional design. The sample size is only medium and therefore, in light of recent discussions of an optimal sample size in correlational studies suggesting sample sizes approaching *n* = 250 (Schönbrodt and Perugini, 2013), should a further validation aim at a size at least twice as large. Additionally, the sample mostly consisted of young participants of academic background (mostly psychology students), it is a convenient sample, whereas no snowballing sampling technique was explicitly used. This, not only offering a limited amount of variance regarding the measured constructs but also decreases the transferability of our findings to other populations. Thus, further studies are needed to test whether the sample is fully representative. However, on one hand, we consider that publishing the German version of the FREE-scale at this point with a first validation will trigger further, highly powered investigations into its psychometric properties. On the other hand, as our CFA results largely match the previously proposed structure (Burton and Bonanno, 2016; Chen et al., 2018), the generalizability of the results may be ensured. Moreover, the sensitivity power analysis indicated that with our sample size we are able to detect effects with considerable power. A further limitation is that the sample composition was mostly conformed by females (77.9%), thus, despite no differences in demographics between females and males who were attested, this information should be taken into account when interpreting the results and designing new studies based on the current findings. Moreover, the reported analyses were based on self-reports data, which may include possible unknown reporting biases and, more generally, might introduce a common method bias that affects the correlations between EF and indices of mental health and well-being that also are based on self-reports.

A final limitation is that heart rate variability measures were only available for a small group of participants (*N* = 68), which does not provide enough statistical power to take into account how HRV may relate to emotion regulation abilities (measured with the FREE scale). Given that HRV is related to the body’s state of balance and stress, further studies are needed to address this question.

Future studies shall aim at investigating the importance of individual differences in flexible emotion regulation, particularly suppression abilities, in influencing cognitive and affective disorders. Additionally, an exploration of how different factors, including individual differences in cognitive abilities (e.g., working memory capacity and cue-guided behavior) are interrelated to the specific individual’s behavior according to task demands would be essential to shed light on strategies that guide behavior toward the most convenient choice.

As already suggested, future studies shall further tackle how flexible emotion regulation abilities depend upon brain regions and networks and how this knowledge can inform the current theoretical models and apply them to investigate these phenomena across the lifespan.

## Conclusions

Here, we provide the German version of the Flexible Regulation of Emotional Expression scale and demonstrate a similar internal factor structure and construct validity as the original version (Burton and Bonanno, 2016). More importantly, we evidence that the FREE-scale may be used as a tool for the investigation of emotional expressive flexibility regarding switching between enhancing and suppressing emotional expressions in response to the situational context. Our results demonstrate that the FREE-scale allows the assessment of emotional flexible regulation abilities and relates them to psychopathological symptoms. This, may, in turn, be applied to investigate the impact of unexpected conditions that restrict personal contact and may offer starting points for evaluating individual characteristics for affective disorders and the development of personalized therapeutic interventions.

## Data Availability Statement

The datasets presented in this study can be found in online repositories. The names of the repository/repositories and accession number(s) can be found below: https://github.com/GGonEsc/EmotionalFlexibilityScale_PaperCode https://osf.io/p92ry/.

## Ethics Statement

The studies involving human participants were reviewed and approved by The institutional review board of the Technische Universität Dresden (EK 227052019). The patients/participants provided their written informed consent to participate in this study.

## Author Contributions

GG-E, SG, and DD contributed to manuscript conceptualization. GG-E analyzed the data and wrote the original manuscript draft. GG-E, SG, GB, and DD contributed to data interpretation and provided critical revisions. DD performed data collection and curation. GG-E, DD, MB, and JT contributed to the translation of the FREE-scale. All authors contributed to the article and approved the submitted version.

## Conflict of Interest

The authors declare that the research was conducted in the absence of any commercial or financial relationships that could be construed as a potential conflict of interest.

## Publisher’s Note

All claims expressed in this article are solely those of the authors and do not necessarily represent those of their affiliated organizations, or those of the publisher, the editors and the reviewers. Any product that may be evaluated in this article, or claim that may be made by its manufacturer, is not guaranteed or endorsed by the publisher.
