# Supplementary material for: Associating Flexible Regulation of Emotional Expression With Psychopathological Symptoms
Source: Front Behav Neurosci. 2022 Jun 27;16:924305. doi: 10.3389/fnbeh.2022.924305 (PMC9272006; doi:10.3389/fnbeh.2022.924305)
Supplement: Supplementary file 1 [file Data_Sheet_1.pdf]

The Flexible Regulation of Emotional Expression (FREE) Scale, German Version

Messung der Fähigkeit, emotionalen Ausdruck zu verstärken und zu unterdrücken

von C. L. Burton und G. A. Bonanno, 2016, Psychological Assessment, *28 (8), 929-941*

http://doi.org/10.1037/pas0000231

FREE (DE: Flexible Regulation des emotionalen Ausdrucks)
Emotionen zu zeigen ist ein fester Bestandteil unseres täglichen Lebens. Aus sozialen Gründen müssen wir manchmal mehr Emotionen ausdrücken als wir fühlen, und manchmal müssen wir weniger Emotionen zeigen als wir fühlen.

Die folgenden Szenarien beinhalten POSITIVE Emotionen. Geben Sie für jedes Szenario an, wie gut Sie in der Lage wären, Ihre Emotionen noch STÄRKER AUSZUDRÜCKEN, als Sie normalerweise fühlen würden:

	**unfähig**					**sehr fähig**
1) Ein Freund/Eine Freundin gewinnt eine Auszeichnung für eine Sportart, die Sie nicht interessiert.	1	2	3	4	5	6
2) Ein Kollege/Eine Kollegin bekommt eine Beförderung und möchte darüber reden.	1	2	3	4	5	6
3) Ein Freund/eine Freundin spricht über ein tolles Date, das sie vor Kurzem hatte.	1	2	3	4	5	6
4) Ein Familienmitglied schenkt Ihnen ein Hemd, das Ihnen nicht gefällt.	1	2	3	4	5	6

Die folgenden Szenarien beinhalten NEGATIVE Emotionen. Geben Sie für jedes Szenario an, wie gut Sie in der Lage wären, Ihre Emotionen noch STÄRKER AUSZUDRÜCKEN, als Sie normalerweise fühlen würden:

	**unfähig**					**sehr fähig**
5) Ein Freund/Eine Freundin erzählt Ihnen, was für einen schrecklichen Tag er oder sie hatte.	1	2	3	4	5	6
6) Ihr Chef/Ihre Chefin beschwert sich über ein Projekt, über das Sie wenig wissen und mit dem Sie nichts zu tun haben.	1	2	3	4	5	6
7) Ein Freund/eine Freundin spricht über eine Trennung, die Sie insgeheim gutheißen.	1	2	3	4	5	6
8) Sie gehen zur Beerdigung von jemandem, den Sie nicht kennen.	1	2	3	4	5	6

Die folgenden Szenarien beinhalten POSITIVE Emotionen. Geben Sie für jedes Szenario an, wie gut Sie in der Lage wären ZU VERBERGEN, wie Sie sich normalerweise fühlen würden:

	**unfähig**					**sehr fähig**
9) Während Sie mit einem Freund/einer Freundin zu Abend essen, der/die gerade den Job verloren hat, erhalten Sie einen Anruf von Ihrem Chef, in dem er Ihnen mitteilt, dass Sie eine Gehaltserhöhung erhalten.	1	2	3	4	5	6
10) Sie nehmen an einem Vortrag teil und sehen einen versehentlichen witzigen Tippfehler in der Diashow des Moderators.	1	2	3	4	5	6
11) Sie sind ein Gast bei einer feierlichen religiösen Zeremonie und die Person, die neben Ihnen sitzt, hat gerade einen lustigen Witz geflüstert.	1	2	3	4	5	6
12) Während eines Treffens mit einem Vorgesetzten beginnt sein/ihr Telefon unerwartet einen peinlichen Klingelton zu spielen.	1	2	3	4	5	6

Die folgenden Szenarien beinhalten NEGATIVE Emotionen. Geben Sie für jedes Szenario an, wie gut Sie in der Lage wären ZU VERBERGEN, wie Sie sich normalerweise fühlen würden:

	**unfähig**					**sehr fähig**
13) Sie befinden sich in Gesellschaft und die Person, mit der Sie sprechen, spuckt während des Sprechens häufig.	1	2	3	4	5	6
14) Sie haben unmittelbar vor einem wichtigen Arbeitstreffen vom Tod eines nahen Verwandten erfahren.	1	2	3	4	5	6
15) Sie sind bei einem ersten Date zum Abendessen in einem Restaurant und ein Fremder verschüttet sein Getränk über Ihnen.	1	2	3	4	5	6
16) Nach einem sehr irritierenden und stressigen Tag kommt ein manchmal nerviger Nachbar vorbei, um Hallo zu sagen.	1	2	3	4	5	6

REFERENZ

English Version:

Burton, C. E., & Bonanno, G. A. (2016). Measuring the Ability to Enhance and Suppress Emotional Expression: The Flexible Regulation of Emotional Expression (FREE) Scale. Psychological Assessment, 28(8), 929-941.
Link zum Paper: http://doi.org/10.1037/pas0000231

German Version:

Gonzalez-Escamilla, G., Dörfel, D., Becke, M., Trefz, J., Bonanno, G.A., & Groppa, S. (2022). Flexible regulation of emotional expressions – Validation of the FREE scale and associations with psychopathology symptoms. Frontiers in Behavioral Neuroscience.
Link zum Paper: http://doi.org/10.3389/fnbeh.2022.924305

AUSWERTUNG

Volle Subskalen: Addieren Sie die Werte innerhalb jeder Subskala, d.h. Positiv-Expressiv („Steigern“ positiver Emotionen), Negativ-Expressiv („Steigern“ negativer Emotionen), Positiv-Verbergen („Verbergen“ positiver Emotionen), Negativ-Verbergen („Verbergen“ negativer Emotionen).

Subskalen „Steigern“ (A) und „Verbergen“ (B): Rechnen Sie die positiven und negativen Expressiv-Subskalen für die Gesamtskala „Steigern“ zusammen und kombinieren Sie die positiven und negativen Unterdrückungsmaßstäbe für eine Gesamtskala „Verbergen“.

Flexibilität: Es gibt zwei Methoden zur Berechnung einer flexiblen Gesamtpunktzahl:

1. Die einfachste Methode ist:
   a. Berechnen Sie den Durchschnitt für jede Skala, A und B
   b. Berechnen Sie die Summe der beiden Mittelwerte (A + B)
   c. Berechnen Sie die "Polarität" als den absoluten Wert der Differenz der beiden Mittelwerte |A – B|
   d. Flexibilität ist die Summe minus der Polarität oder (A + B) – |A – B|
2. Eine zweite, etwas kompliziertere Methode verwendet negative Beschleunigung. In dieser Methode:
   a. Berechnen Sie den Durchschnitt für jede Skala und bestimmen Sie den größeren Durchschnitt (L) und den kleineren Durchschnitt (S).
   b. Flexibilität ist dann [(2S + 1) – (S + L +2)].
   c. Wenn die beiden Mittelwerte gleich sind, dann geben Sie entweder L und S an und führen Sie die Berechnung aus

Diese beiden Methoden erzeugen fast identische (d. H. Sehr stark korrelierte) Werte. Die erste Methode bietet eine grundlegende Flexibilität Punktzahl und wurde in der Mehrzahl der Publikationen unseres Teams verwendet. In einigen Fällen führt die erste Methode jedoch zu ungleichmäßigen Inkrementen zwischen den Flexibilitätsstufen. Die zweite Methode verwendet negative Beschleunigung, um etwas glattere Inkremente zu erstellen. Jedoch ist die negative Beschleunigungsmethode weniger effektiv, wenn die Daten eine begrenzte Variabilität oder einen begrenzten Bereich haben, im solche Fall wird die erste Methode empfohlen.
