# Supplementary material for: Associating Flexible Regulation of Emotional Expression With Psychopathological Symptoms
Source: Front Behav Neurosci. 2022 Jun 27;16:924305. doi: 10.3389/fnbeh.2022.924305 (PMC9272006; doi:10.3389/fnbeh.2022.924305)
Supplement: Supplementary file 2 [file Data_Sheet_2.pdf]

Supplemental Materials

Measuring Ability to Enhance and Suppress Emotional Expression: The Flexible Regulation of Emotional Expression (FREE) Scale

by C. L. Burton & G. A. Bonanno, 2016, *Psychological Assessment, 28(8),* 929-941

http://dx.doi.org/10.1037/pas0000231

FREE (Flexible Regulation of Emotional Expression)

Displaying emotion is a regular part of our daily lives. For social reasons, sometimes we have to express more emotion than we are feeling, and sometimes we have to display less emotion than we are feeling.

The following scenarios involve POSITIVE emotion. For each scenario, indicate how well you would be able to be even MORE EXPRESSIVE than usual of how you were feeling:

	Unable					Very able
1) A friend wins an award for a sport that doesn't interest you.	1	2	3	4	5	6
2) A coworker gets a promotion and wants to talk about it.	1	2	3	4	5	6
3) A friend is talking about a great date she had the other night.	1	2	3	4	5	6
4) You receive a gift from a family member but it's a shirt you dislike.	1	2	3	4	5	6

The following scenarios involve NEGATIVE emotion. For each scenario, indicate how well you would be able to be even MORE EXPRESSIVE than usual of how you were feeling:

	Unable					Very able
5) Your friend is telling you about what a terrible day they had.	1	2	3	4	5	6
6) Your boss is complaining about a project you know little about and have no involvement with.	1	2	3	4	5	6
7) A friend is talking about a break-up that you secretly think is a good thing.	1	2	3	4	5	6
8) You're attending the funeral of someone you don't know.	1	2	3	4	5	6

The following scenarios involve POSITIVE emotion. For each scenario, indicate how well you would be able to CONCEAL how you were feeling:

	Unable					Very able
9) While having dinner with a friend who has just recently lost their job, you receive a phone call from your boss stating you will get a raise.	1	2	3	4	5	6
10) You are in a training session and you see an accidentally funny typo in the presenter's slideshow.	1	2	3	4	5	6
11) You're a guest at a solemn religious ceremony and the person sitting next to you just whispered a funny joke.	1	2	3	4	5	6
12) During a meeting with a supervisor, his/her phone unexpectedly begins to play an embarrassing ringtone.	1	2	3	4	5	6

The following scenarios involve NEGATIVE emotion. For each scenario, indicate how well you would be able to CONCEAL how you were feeling:

	Unable					Very able
13) You are at a social event and the person you're talking to frequently spits while they speak.	1	2	3	4	5	6
14) You have just heard about the death of a close relative right before an important work meeting.	1	2	3	4	5	6
15) You are on a first date at a restaurant having dinner, and a stranger spills their drink on you.	1	2	3	4	5	6
16) After you have a very irritating and stressful day, a sometimes-annoying neighbor stops by to say hello.	1	2	3	4	5	6

CITATION

Burton, C. E., & Bonanno, G. A. (2016). Measuring the Ability to Enhance and Suppress Emotional Expression: The Flexible Regulation of Emotional Expression (FREE) Scale. *Psychological Assessment, 28(8),* 929-941.

View article:

http://dx.doi.org.ezproxy.cul.columbia.edu/10.1037/pas0000231

Files:

z1t004152979so1.doc

SCORING:

Full subscales: total each subscale for positive and negative enhance and suppress scales. Enhance and suppress subscales: Combine the positive and negative enhance scales for overall enhance scale, and combine the positive and negative suppress scales for an overall suppress scale.

Flexibility: There are two methods for calculating an overall flexibly score:

1. The simplest method is to:
   a. Calculate the average for each scale, A and B
   b. Calculate the "sum" the two averages (A + B)
   c. Calculate the "polarity" as the absolute value of the difference of the two averages [A - B]
   d. Flexibility is the sum minus the polarity or (A + B)-[A - B]
2. A second, slightly more complicated method uses negative acceleration. In this method:
   a. Calculate average for each scale and determine the larger average (L) and smaller average (S).
   b. Flexibility is then [(2S + 1)/(S + L +2)].
   c. If the two averages are equal, then designate either as the L and S and run the calculation

These two methods produce almost identical (i.e., very highly correlated) scores. The first method provides a basic flexibility score and has been used in the majority of publications from our team. Under some circumstances, however, the first method leads to unequal increments between levels of flexibility. The second method uses negative acceleration to create somewhat smoother increments. However negative acceleration method is less effective when data have limited variability or range, in which case the first method is suggested.
